# Antibacterial Activity of Glucomoringin Bioactivated with Myrosinase against Two Important Pathogens Affecting the Health of Long-Term Patients in Hospitals

**DOI:** 10.3390/molecules181114340

**Published:** 2013-11-20

**Authors:** Maria Galuppo, Gina Rosalinda De Nicola, Renato Iori, Pia Dell’Utri, Placido Bramanti, Emanuela Mazzon

**Affiliations:** 1 IRCCS Centro Neurolesi “Bonino-Pulejo”, Via Provinciale Palermo, S.S.113, Contrada Casazza, Messina 98124, Italy; E-Mails: mariagaluppo@gmail.com (M.G.); piadellutri@gmail.com (P.D.); bramanti.dino@gmail.com (P.B.); 2 Consiglio per la Ricerca e la sperimentazione in Agricoltura, Centro di Ricerca per le Colture Industriali (CRA-CIN), Via Di Corticella 133, Bologna 40128, Italy; E-Mail: g.denicola@isci.it

**Keywords:** glucomoringin, isothiocyanate, *Moringa oleifera*, antibiotic activity, *Staphylococcus aureus*, *Enterococcus casseliflavus*

## Abstract

Glucosinolates (GLs) are natural compounds present in species of the order *Brassicales* and precursors of bioactive isothiocyanates (ITCs). In the recent years, they have been studied mainly for their chemopreventive as well as novel chemotherapeutics properties. Among them 4-(α-L-rhamnosyloxy)benzyl glucosinolate (glucomoringin; GMG), purified from seeds of *Moringa oleifera* Lam., a plant belonging to the *Moringaceae* family, represents an uncommon member of the GL family with peculiar characteristics. This short communication reports new evidences about the properties of GMG and presents a new innovative utilization of the molecule. The bioactivation of GMG by myrosinase enzyme just before treatment, permits to maximize the power of the final product of the reaction, which is the 4-(α-L-rhamnosyloxy)benzyl isothiocyanate (GMG-ITC). We tested the antibiotic activity of this latter compound on two strains of pathogens affecting the health of patients in hospital, namely *Staphylococcus aureus* and *Enterococcus casseliflavus*, and on the yeast *Candida albicans*. Results show that the sensibility of *S. aureus* BAA-977 strain and *E. casseliflavus* to GMG-ITC treatment reveals an important possible application of this molecule in the clinical care of patients, more and more often resistant to traditional therapies.

## 1. Introduction

**Scheme 1:**
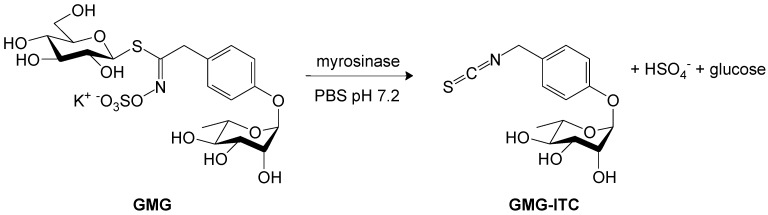
Production of 4-(α-L-rhamnosyloxy)benzyl isothiocyanate (GMG-ITC). Myrosinase-catalyzed hydrolysis reaction of glucomoringin (GMG), purified from *Moringa oleifera* seeds, in phosphate buffered saline (PBS) solution (pH 7.2) to produce GMG-ITC.

The aim of this work was to extend and consolidate some well known applications of a naturally occurring compound present in *Moringa oleifera* Lam. in the treatment of the most common clinical infections. Seeds of the plant contain 8%–10% of a structurally unusual glucosinolate (GL), the compound 4-(α-L-rhamnosyloxy)benzyl GL (glucomoringin; GMG). Chemically, GMG presents a unique characteristic structure consisting of an additional saccharidic residue in its side chain [1]. GMG is a typical secondary metabolite present in plants belonging to the *Moringa* genus that consists of 14 species, among which *M. oleifera* (horseradish tree) is the most widely distributed. *M. oleifera* is a multipurpose tree which grows in many tropical or equatorial regions, around dry tropical areas in Northwestern India at the Southwestern foot of the Himalaya, that is used for human and animal nutrition, and medicinal purposes [2]. Owing to its bactericidal properties the plant is also currently employed for water purification [3]. The medical value of the seeds and other parts of the plant has long been recognized in folk medicine and different extracts have also been tested as anticancer, anti-inflammatory, and hepato-protective agents [4,5], and for their antimicrobial activity [6,7]. These properties are mainly attributed to the glycosylated isothiocyanate (ITC), 4-(α-L-rhamnosyloxy)benzyl ITC (GMG-ITC), resulting from myrosinase (β-thioglucoside glucohydrolase; E.C. 3.2.1.147) hydrolysis of GMG (Scheme 1). The ready availability of pure GMG, purified from *M. oleifera* seeds with myrosinase enzyme at CRA-CIN (Bologna, Italy) suggested us to exploit a new therapeutic way for treating two important pathogens affecting the health of patients in a medical centre. Bioactive GMG-ITC can be obtained quickly starting from a phosphate buffered saline (PBS) solution of pure GMG by adding myrosinase just before treatment in order to maximize the power of the final product and avoiding the poor solubility of this ITC. The effort to discover new therapeutic methods at our Institute of Hospitalization and Care has led us to evaluate the possibility that bioactive GMG-ITC could counteract microbial growth, in particular that of *Staphylococcus aureus* (*S. aureus*) and *Enterococcus casseliflavus* (*E. casseliflavus*)—two important pathogens affecting the health of patients in public institutions—and at the same time, provide an evaluation on the possible antimycotic power towards a *Candida albicans* (*C. albicans*) strain. To date, methicillin-resistant *S. aureus* and multiresistant enterococci constitute a serious and real nosocomial infection problem [8], so new challenges have emerged to counteract them. In this study we performed *in vitro* assays to assess the antibiotic ability of GMG bioactivated with myrosinase against the Gram positive bacteria *S.* *aureus* and *E. casseliflavus*, and against *C. albicans*, one of the most common yeasts that causes serious infections. We have obtained interesting preliminary data that suggest a new application of this safe and effective product in the future.

## 2. Results and Discussion

GL hydrolysis is the most used method to obtain ITCs as reaction product. Differently from the current literature, the main innovation of the present work is the use of an ITC, the named GMG-ITC, according to a procedure that provides not an alcoholic extract, such as other authors report testing a methanol and *n*-hexane seed extract of *Moringa oleifera* and *Moringa stenopetala* (please see Atieno *et al.* for more details [9]), but rather a soluble, highly pure product.

Moreover, in a previous study performed in 1991, the *in vitro* antimicrobial activities of *M. oleifera* leaves, roots, bark and seeds were investigated against bacteria, yeast, dermatophytes and helminths pathogenic to humans [10]. The results of this study demonstrated that fresh leaf juice, as well as aqueous extracts of the seeds, inhibit the growth of *Pseudomonas aeruginosa* (*P. aeruginosa*) and *S. aureus*, whereas no activity was found against four other pathogenic Gram-positive and Gram-negative bacteria, such as against *C. albicans*.

Our current aim was to perform *in vitro* studies to assess the antibiotic capability of the new composition of GMG bioactivated with myrosinase (Scheme 1) against some Gram positive bacteria and the most common yeast that causes serious infections, *C. albicans*.

Among the major human harmful bacteria pathogens, responsible of a wide spectrum of diseases, *S. aureus* is one of the most widespread causing successful pandemics [11,12]. Its ability to cause a diverse array of life-threatening infections and its capacity of adaptation under different environmental conditions has also let to face the serious problem of drug-resistance. Thus, this organism constitutes an important public health burden, not only in the hospital environment but also in the community setting [13]. In this perspective, about more than five decades ago, methicillin-resistant *S. aureus* (MRSA) emerged as a clinically relevant human pathogen. Hospitals and other health care facilities are a favourable environment for dissemination of this virulent pathogen, due to the vulnerability of patients and the frequent exposure to intensive antimicrobial therapy [14].

**Figure 1:**
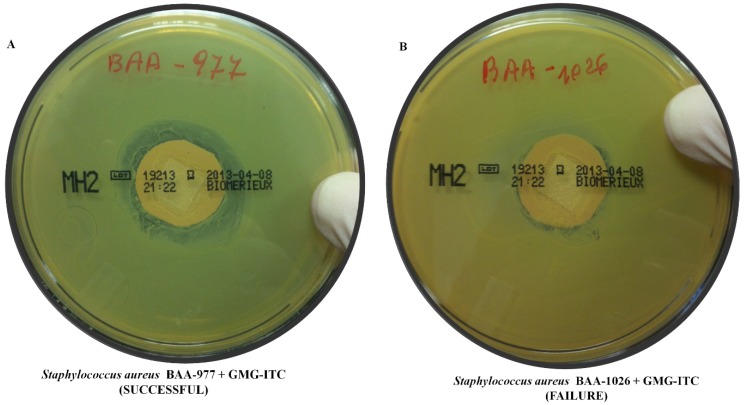
Antibiogram to assess *S. aureus* sensibility to GMG-ITC treatment. To verify the sensibility of *S. aureus* to GMG-ITC, BAA-977 strain and BAA-1026 strain were incubated for 24 h with the antibiotic according to a disk-diffusion method. Results indicate that GMG-ITC inhibits the bacterial growth of BAA-977 strain (**A**), with an inhibition halo of about 25 ± 1 mm, but it was not able to counteract the bacterial growth of the strain BAA-1026 (**B**).

Due to the importance of discovering new antibiotics in the treatment of infections by *S. aureus*, we tested the possible antibacterial activity of the purified GMG, obtained from *M. oleifera* seeds, and bioactived with myrosinase to obtain GMG-ITC. Incubation of bacteria with bioactive GMG-ITC for 24 h shows that this compound is able to inhibit bacterial growing of BAA-977 (Figure 1A) with an inhibition halo of about 25 ± 1 mm of diameter, while it results weakly or not effective against BAA-1026 strain (Figure 1B). Confirming these data, a normal growth was noted when bacterial plates were incubated with PBS, vehicle of GMG, while a growth inhibition there was when plates were incubated with vancomycin (data not shown).

The susceptibility of the *S. aureus* against GMG-ITC suggested us to investigate whether this compound could have some effects on another Gram positive bacteria. For this reason, we tested the effect of GMG-ITC plating *E. casseliflavus* in the presence of an antibiotic for 24 h. This bacterium was chosen because increasingly important nosocomial and community-acquired pathogens are more often due to enterococci colonization. In fact, although they are generally considered to be of low pathogenic potential, it is now well recognized that these organisms can cause serious invasive infections, including endocarditis, bacteremia, urinary tract and pelvic infections [15].

**Figure 2:**
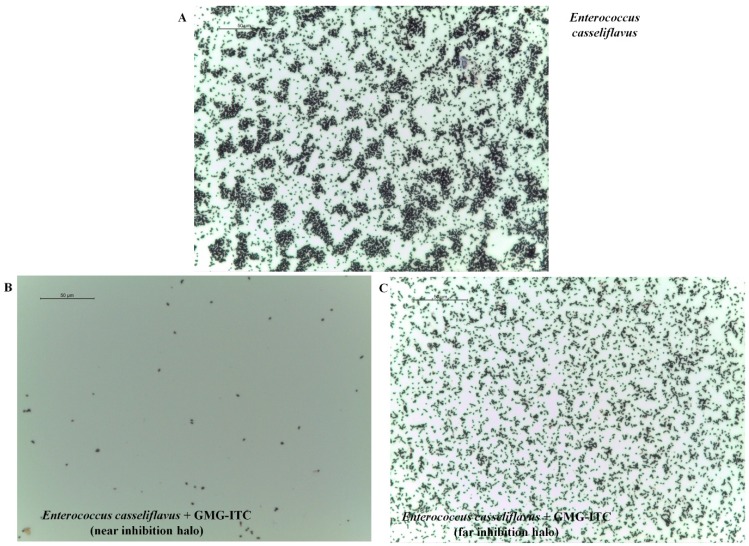
*E. casseliflavus* susceptibility to GMG-ITC treatment. *E. casseliflavus* was incubated with GMG-ITC for 24 h and a loopful near and far the inhibition halo was taken to perform Gram staining. The results show that a very low bacterial growth is present in correspondence of the inhibition halo (**B**). Moreover a higher bacterial concentration has been detected far from the inhibition halo (**C**) but, anyway, a lower number of bacterial per cm^2^ was detected in comparison to plates where antibiotic treatment was not applied (**A**). Images were taken using a 40× objective, scale bar = 50 µm.

Particularly virulent strains of *Enterococcus*, resistant to conventional antibiotic treatment, have emerged in nosocomial infections of hospitalized patients, especially in the US. More specifically, of particular interest is the increasingly presence of vancomycin-resistant enterococci (VRE), that lead to infections of the urinary tract associated to the use of catheters or to bloodstream infections related to vascular catheters [16]. Several studies have demonstrated that *E. casseliflavus* is a colonizer of the gastrointestinal tracts of hospitalized patients, nonetheless the virulence of this bacterium should not be underestimated since a case of enterococcal meningitis caused by *E. casseliflavus* was also reported [17]. Our study demonstrated that, at the end of the 24 h, there was an evident inhibition halo (data not shown), and a loopful near and far the area of the halo was taken and submitted to Gram staining. The image in Figure 2 shows evident absence of *E. casseliflavus* growth around the patch soaked with GMG bioactivated with myrosinase (Figure 2B). A higher rate of bacterial growth was detected in the distal area (Figure 2C), but, in any case, the percentage of bacteria was lower than the one recorded in the control plate where *E. casseliflavus* was seeded in the absence of GMG-ITC (Figure 2A).

**Figure 3:**
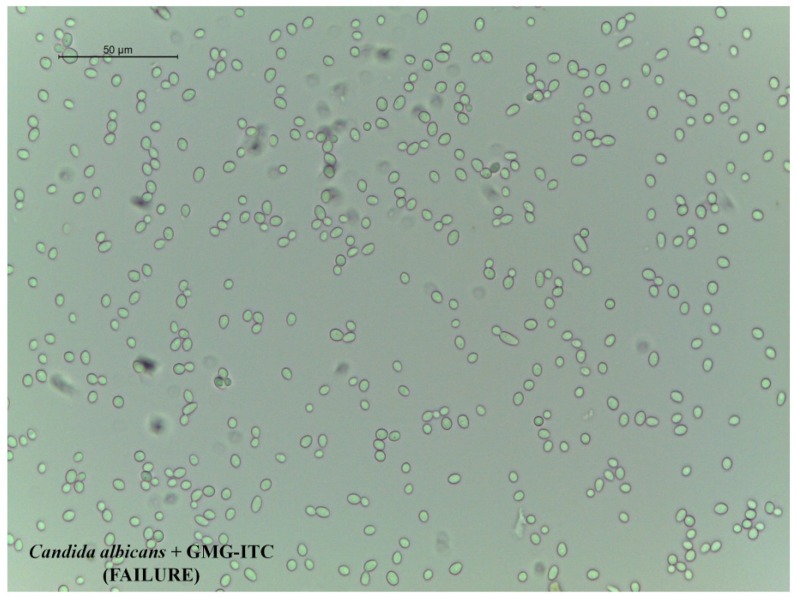
*C. albicans* resistance to GMG-ITC treatment. Studies performed to assess *C. albicans* sensibility to GMG-ITC reveal that the molecule does not have any effecton this yeast. In the figure, thefailure of the treatment has been reported. Image was taken using a 40× objective, scale bar = 50 µm.

Finally, our efforts were aimed to evaluate the possible activity against *C. albicans* yeast but, conversely, GMG-ITC treatment was not able to counteract the *in vitro* growth of this pathogen (Figure 3), confirming the failures previously reported by other authors [10].

## 3. Experimental

### 3.1. GMG and Myrosinase Purification

GMG was isolated from *M. oleifera* Lam. (fam. *Moringaceae*) seeds (cake powder PKM2 provided by Indena India Pvt. Ltd.; Bangalore, India) according to a previously described method [18]. In brief, GMG was purified by two sequential chromatographic steps: isolation through anion exchange chromatography, followed by gel filtration to achieve purification to homogeneity. GMG was unambiguously characterized by ^1^H- and ^13^C-NMR spectrometry and the purity was assessed by HPLC analysis of the desulfo-derivative according to the ISO 9167-1 method [19], yielding GMG with a purity of 99% based on peak area value, and about 90%–92% on a weight basis due to its highly hygroscopic properties. The enzyme myrosinase was isolated from seeds of *Sinapis alba* L. as described by Pessina *et al*. [20] with some modification. The specific activity of the stock solution used in the present study was 60 U/mg of soluble protein. The enzymatic activity was 32 U/mL and the solution was stored at 4 °C in sterile saline solution at neutral pH until use. One myrosinase unit was defined as the amount of enzyme able to hydrolyze 1 µmol/min of sinigrin at pH 6.5 and 37 °C [21].

### 3.2. Enzyme Bioactivation of GMG and Patch Application

GMG powder (1.52 mg/mL) was dissolved in PBS solution pH 7.2 and bioactived with myrosinase (30 µL for one mL of GMG solution) for 15 min with the aim of obtaining the bioactive GMG-ITC quickly. The total conversion of pure GMG into GMG-ITC was confirmed by HPLC analysis of the GMG desulfo-derivative, which allowed us to monitor the reduction of GMG until its complete disappearance in the reaction mixture [19]. All bacterial strains and agar plates were provided by bioMérieux SA (Marcy l’Étoile - France).

Two separate plates of Mueller-Hinton 2 agar were used to test susceptibility of two strains of *S. aureus* (*Staphylococcus aureus* Rosenbach ATCC^®^ BAA-1026 and *Staphylococcus aureus* subsp. *aureus* Rosenbach ATCC^®^ BAA-977). Petri dishes were routinely seeded and incubated for 24 h with a patch soaked of the bioactive GMG-ITC solution.

According to the same protocol described before, *E. casseliflavus* strain (ATCC^®^ 700327) in blood-agar plates, and *C. albicans* yeast (ATCC^®^ 14053) in Sabouraud Gentamicin Chloramphenicol 2 agar, were seeded and covered with a patch soaked of the GMG-ITC solution for 24 h. Moreover, with regard to *E. casseliflavus*, a loopful near and far inhibition halo was evaluated performing Gram staining.

A positive control given by bacterial plates incubated with conventional antibiotic therapy (no growth) and negative control given by bacterial plates incubated with PBS (normal growth) were provided. All results are representative of three separate set of experiments. Data are shown as average antibacterial activity mean ± S.E.M.

## 4. Conclusions

Currently, there is an increasing interest worldwide aimed at identifying bioactive compounds from plant sources, such as *M. oleifera*, that can be pharmacologically effective with low or no side effects [22]. Purified GMG together with myrosinase, which releases bioactive GMG-ITC, constitutes an innovative and effective antibiotic with greater advantages compared to ethanol or aqueous extracts of *M. oleifera* leaves. We have demonstrated that GMG bioactivated with myrosinase inhibits *S. aureus* growth. Moreover, we have given new evidences for other strain of Gram positive bacteria, in particular *E. casseliflavus*, of being inhibited by GMG-ITC. The preparation of the phytochemical presented here represents a new promising application for the clinical practice in the treatment of nosocomial infections that could help to overcome failures due to the occurrence of antibiotic-resistance. Conversely, the *in vivo* efficacy of the bioactive GMG-ITC preparation on *C. albicans* colonies was not demonstrated and supported.
